# Risk Constraints in the Context of Big Data and Optimization of a Virtuous Interaction Mechanism for Insurance Management Based on Distorted Risk Metrics

**DOI:** 10.1155/2022/2343181

**Published:** 2022-09-06

**Authors:** Yuanyuan Zhao

**Affiliations:** Jiumaojiu International Holdings Limited, Guangzhou 510000, China

## Abstract

A relevant definition of big data technology has been released by relevant research institutes in China. Big data technology is defined as follows: big data technology is a digitally important productivity factor in the network era; big data technology is based on network technology, statistical technology, and mathematical technology; big data technology, as a carrier of network technology, should be integrated with various industries and technologies other than contemporary ones in order to achieve better informatization and industrialization. In the context of the new crown epidemic in 2020, China's big data technology continues to grow steadily, with a total value of 39.2 trillion yuan, accounting for 38.6% of GDP. This paper investigates the risk constraint in the context of big data and optimizes the benign interaction mechanism of insurance management based on distorted risk metrics in this context. The current situation of insurance risk is investigated through a profound discussion of theories in this paper and through research methods such as distortion risk metrics and SQL Server database management. It is found that the overall situation of insurance industry development in the era of digital economy is not optimistic and so on. And relevant suggestions are made in the conclusion.

## 1. Introduction

A relevant definition of big data technology has been issued by relevant research institutes in China. It defines big data technology as follows: big data technology is an important productivity factor of digitalization in the network era; big data technology is based on network technology, statistical technology, and mathematical technology; big data technology, as a carrier of network technology, should be integrated with various industries and technologies other than contemporary ones in order to achieve better informatization and industrialization. Accelerate industrial progress and urbanization and transform the economic structure [1]. In the context of the new crown epidemic in 2020, China's big data technology continues to grow steadily, with a scale of 39.2 trillion yuan, accounting for 38.6 percent of GDP [2]. In China, a new five-year plan and long-term plan were released at the same time, and big data technology was included in the development of China's plan to promote the comprehensive development of big data, the development of big data into all walks of life, the process of digitalization in China, and the building of digital power. China's Central Political Bureau has conducted a number of comprehensive big data studies in the process of China's big data. Digital technology has been integrated into the development of all aspects of life in China, and the general secretary has repeatedly emphasized the importance of using big data technology to transform traditional industries all the way through [3]. Insurance has natural digital attributes, and big data and technology empowerment can optimize the insurance business process and inject new momentum into the industry's development; at the same time, the insurance industry's big data transformation can provide strong support for China's digital economic system. Exploring the new characteristics of insurance industry development in the era of big data, finding new opportunities for insurance industry development in the big data economic system, and studying the dilemmas and challenges facing the development of big data transformation of insurance industry are important in this context for promoting the industrialization of big data economy, accelerating insurance industry reform, and hastening the deep integration of insurance industry [4].

## 2. Research Background

Domestic research on the development of the insurance industry in the context of big data is as follows: Roland et al. point out that the competition in the insurance industry in the era of big data is based on the degree of understanding of customers and product supply [5]. Serkan and Fatih analyze that the big data transformation of insurance enterprises is mainly reflected in informatization, automation, and scenarioization and point out five issues that are concerned about the big data transformation of insurance enterprises [6]. Asier et al. examine how big data technology drives the changes in consumers' demand for insurance from the perspective of people's consumption behavior and give suggestions on how insurance companies can respond to these changes [7]. Li et al. provide three digital transformation realization paths for insurance companies, including optimizing customer experience, optimizing operation models, and innovating business models, from the essential characteristics of big data technology [8]. By introducing the development experiences of InsurTech enterprises in the United States, Singapore, Germany, and other regions, Lukas et al. put forward the suggestions of government support, social participation, and collaboration among all parties to promote the development of insurance enterprises and InsurTech in China [9].

From the perspective of foreign scholars, Hisako et al. suggest that the entry threshold of the insurance industry will be lowered in the era of big data, more competitive entities will come in, and insurance companies must reposition the value of customers and their core competitiveness in order to occupy a place in the competition [10]. Reimund and Oleksandr believe that insurance companies are providers of risk services and should make full use of the Internet to provide efficient and convenient solutions and risk management for their customers [11]. Sidorova et al. believe that the development of the Internet has influenced the direction of business in the insurance industry [12]. De Lei et al. state that making underwriting and claims online through the Internet can greatly improve efficiency and enhance user experience compared to the traditional model [13]. Muhamat et al. point out that one of the major impacts of the development of online insurance on the insurance industry is that it is more convenient for customers to compare prices, and insurance companies should not see this change as a profit threat but should think about how to change their business model [14].

According to the above analysis, scholars both at home and abroad have focused on the positive aspects of the development of the insurance industry in the context of big data and have suggested that insurance companies should transform in line with the trend, but there has been little research on the specific situation of the development of the insurance industry in China under the macro environment of big data and the dilemma brought about by the emergence of new technologies. However, only by studying the characteristics of digital technologies and analyzing them in combination with the development status and policy background of China's insurance industry today can we better discover how digital technologies should be integrated into various aspects of the insurance industry and give full play to their advantages, as well as digging deeper into the challenges that may be faced to help China's insurance industry ride the wave of change in the new era.

## 3. Materials and Methods

### 3.1. Basic Theory

#### 3.1.1. Big Data Collection and Management

**Figure 1:**
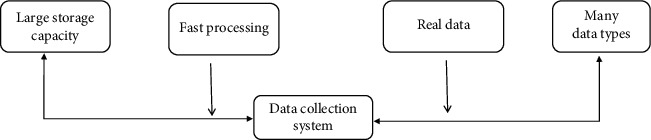
Characteristics of the data collection system.

This paper's research content is a discussion of the network literacy education system for college students under the big data media management mode. As a result, a big data collection and management system is required, which is used today not only to store a large amount of data and form a large number of data storage network systems but also to analyze and process data extremely quickly [15]. Big data management systems are all about analyzing and processing a wide variety of data with a reasonable use of media such as computers and networks. It is becoming more and more popular in all aspects of life with fast and convenient digital information transmission and processing, bringing a great degree of information convenience to people's future learning life, updating people's traditional view of data management, providing a more innovative and convenient way to store and process complicated data, and greatly improving people's work efficiency [16]. The big data management system has four main features such as large storage volume, rapid information processing, real and effective data results, and a wide variety of data types, as shown in Figure 1.

#### 3.1.2. Insurance Risk Management in the Era of Big Data

**Figure 2:**
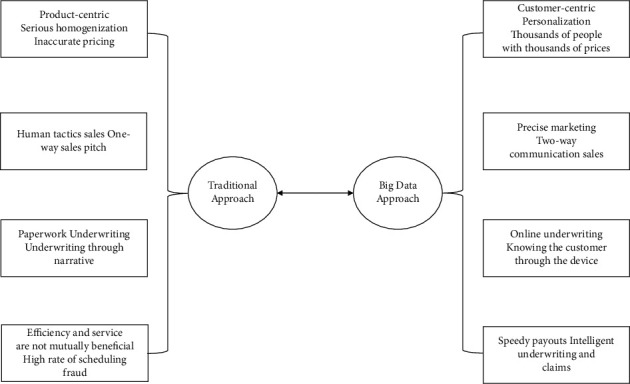
Comparison of the traditional insurance industry and the insurance industry's business development link in the era of big data.

The traditional insurance industry has problems such as single type of insurance products, human sea mode marketing, and long underwriting and claims process due to the limitation of technology. Through the introduction of the above 4 major insurance technology technologies, we can see that the drive of insurance technology in the digital economy era has a great impact on various aspects of the insurance industry such as product development, insurance marketing, insurance underwriting, and insurance claims [16]. The impact of different technologies on different aspects of the insurance industry varies, and a new concept and model of the insurance industry has been formed. The application of insurance technology in the digital economy can improve the existing business pain points and expand the social influence and social value of the insurance industry [17], as shown in Figure 2:

 
*Product Development Link*. The traditional insurance companies are more production-oriented and product-centered in product design, which has problems such as serious product homogenization, small coverage, and inaccurate pricing. The emergence of digital technology will break through the limitations of traditional insurance product design in two aspects: product innovation design and accurate pricing [18]. (1) Product innovation design. From the supply side of the insurance market, although thousands of insurance products have been launched by major insurance companies, only a few dozen of them can really open the market and be favored by consumers. One of the major reasons why insurance companies have difficulty in designing insurance products that are marketable is that the insurance products they design do not have enough depth of integration with customers' daily lives, making it difficult for customers to realize the need for a particular insurance business. For example, Zhong An Insurance uses big data and cloud computing technology to provide return shipping insurance for online shoppers. Ping An General Insurance relies on AI technology to promote the development of pet liability insurance. The “insurance” uses AI and big data technology to provide risk protection for O2O, sharing economy, sports, and other scenarios, increasing the fit between customers' lives and insurance products and insurance needs, and opening up a blue ocean that the traditional insurance market has never ventured into. (2) Accurate pricing of products. Due to information asymmetry, traditional insurance products are unable to make independent risk assessment and product pricing based on customers' personal information, and the emergence and application of digital technology will fundamentally solve this problem. Artificial intelligence devices such as smart watches, smart bracelets, and smart homes enable insurance companies to gain a deeper understanding of consumers' daily behavioral activities. Big data technology categorizes and analyzes the collected data on customers' personal preferences, behavioral habits, and health conditions and can provide differentiated insurance pricing for customers. For example, the “Step Insurance” critical illness insurance jointly launched by Zhong An Insurance and Xiaomi Sports and Le Dynamics APP is based on the number of steps taken by different users as the basis for premium pricing and implements a dynamic pricing mechanism to motivate customers to exercise while reducing the payout rate of insurance companies, achieving a win-win situation. Digital technology has helped insurance companies shift from “product-centric” to “customer-centric.”
 
*Product Marketing*. (1) Achieving precision marketing. Traditional marketing in the insurance industry is mainly sales-oriented, where agents or marketers market existing insurance products to customers, and the information channels for customers to understand insurance products are mainly through the introduction of marketers or their own conscious or unconscious exposure to television and Internet publicity, and if they do not spend some time to understand the relevant information, it is difficult for customers to find insurance products that perfectly match their own desires. The use of big data and artificial intelligence has fundamentally altered the traditional marketing model and enabled product personalization [19]. (2) Broadening the contact channels between customers and insurance. The traditional insurance marketing in the insurance industry is more of one-way marketing, which is carried out by marketers or agents who take the initiative to visit or adopt telemarketing, so that customers do not have enough daily contact with insurance and have limited channels to understand insurance products, which makes some customers have doubts when taking out insurance and disputes to be easily caused when claiming. The development of artificial intelligence technology allows insurance sales to be conducted in the form of online interaction, answering customers' doubts and providing corresponding insurance products, turning insurance sales from one-way sales to two-way communication. The outbreak of the new crown epidemic in 2020 accelerated the migration of insurance sales from offline to online, and the Internet interaction will also promote customers' understanding of insurance products.
 
*Insurance Underwriting Link*. (1) The policy takes effect after the customer signs with an agent or marketer. After entering the digital economy, insurance companies are more likely to use digital platforms to conduct insurance exhibition business and customers to take out insurance policies online. With the further application of artificial intelligence in the underwriting process, facial recognition can be done only by cell phones to speed up the identification of identity information, calculate scientific and reasonable premiums through the information entered and the relevant data of existing customers in the background, and make the decision of underwriting or not, which is significantly better than the traditional offline cumbersome paperwork mode of insurance underwriting. (2) Reduce the risk of adverse selection. The complexity of underwriting insurance products lies in the need to understand the basic information of customers and minimize the risk of adverse selection, so as to control the payout rate and achieve sustainable operation.
 
*Insurance Claims Processing*. (1) Realize efficient underwriting. Insurance underwriting work is an important part of insurance risk control, and meticulous and perfect underwriting work can greatly shorten the claims cycle and reduce the claims complaint rate and also reduce the occurrence of fraudulent insurance incidents and lower the payout rate. The traditional insurance underwriting process in the insurance industry mainly relies on the level of customer integrity and the experience of underwriters, and the tedious and lengthy process of fixing damages and claims reduces the customer experience. For this reason, many insurance companies have made no underwriting, no medical examination, and a very fast claims payment standard for some insurance products, which has led to some fraudulent insurance incidents. Efficiency and service have always been the pain points of the traditional insurance industry in underwriting claims. The emergence of artificial intelligence has led to a shift in this situation. (2) Improving antifraud capabilities. According to the International Association of Insurance Supervisors (IAIS), the lack of data makes it difficult for traditional insurance companies to overcome the problem of insurance fraud caused by information asymmetry, and the restrictive clauses are mainly based on limited fraud models, leading to a series of incidents of malicious insurance fraud. Compared with the tedious manual audit, the two-wheeled platform of “rule audit + big data risk control” built by insurance companies with the help of big data and AI model can accurately capture dozens of typical frauds and abuses of health insurance funds, such as false hospitalization, item cascading, and decomposition of hospitalization, which helps insurance companies reduce medical cost expenses.

### 3.2. Research Method

#### 3.2.1. Distortion Risk Metric

The set (Ω, *ζ*, *P*) is a conceptual space, and *x* is the set of all wandering variables on the space involved. A risk measure *ρ* is a mapping *x* from an *x*_*ρ*_ subset of *R* to the real numbers, denoted as *ρ* : *X* ∈ *x*_*ρ*_↔*ρ*(*X*) ∈ *R*.

First, define the *g* function called the distortion function (distortionfunction) *g* : [0,1]⟶[0,1] if it is a monotonically nondecreasing function and satisfies *g*(0)=0, *g*(1)=1.

Next, define the *ρ*_*g*_ : *x*⟶*R* risk measure often called distortionriskmeasure, if *ρ*_*g*_(*X*) satisfies

(1) $$\begin{array}{c} \rho_{g}\left(X\right)≔\int_{-\infty }^{0}lg\left(S_{X}\left(x\right)\right)-1dx+\overset{\infty }{\underset{0}{\int }}g\left(S_{X}\left(x\right)\right)dx,X\in x, \end{array}$$.

The *X* assumption is that the total risk faced *f* : [0, *∞*)⟶[0, *∞*) by the insurer *f*(*X*) is the partition function, representing the insurer transferring part of the risk faced by itself to the reinsurer. The reinsurer charges the insurer for the insurance premium to supplement the risk they bear because they assume a portion of the insurer's risk. In this paper, we assume that the reinsurance cost criterion has the following form:

(2) $$\begin{array}{c} \mu_{r}\left(f\left(X\right)\right)=\int_{0}^{\infty }r\left(S_{f\left(x\right)}\left(x\right)\right)dx\text{,} \end{array}$$.

Without loss of generality, we assume that *r* is not a function that is zero almost everywhere and that the total risk an insurer has to face is the residual risk it will face plus the cost required to transfer the risk. In terms of a formula, this can be expressed as

(3) $$\begin{array}{c} T_{f}\left(X\right)=X-f\left(X\right)+\mu_{r}\left(f\left(X\right)\right)\text{.} \end{array}$$

#### 3.2.2. SQL Server Big Data Management System

Based on today's mainstream Windows and other operating system platforms, SQL Server database as a new generation of database and analysis of the processing platform software is rapidly being widely used and widely accepted by various enterprise customers. Unlike other current database platforms such as FoxPro and smaller databases such as Access, SQL Server has a complete range of powerful and easy-to-use database management and service processing functions. There are engines that support development, standard database languages such as SQL, and extended features such as replication, OLAP, and analytics. It is also significantly ahead of the rest of the market in terms of other key features that only large database software can have access to [20], such as stored procedures and triggers.

**Figure 3:**
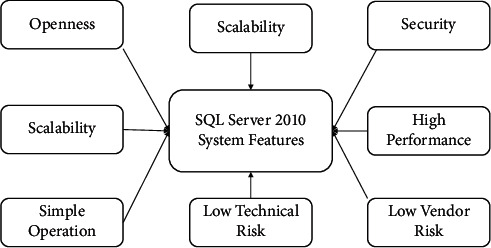
SQL Server 2010 system main features.

Microsoft SQL Server 2010 is based on Microsoft SQL Server 7.0, greatly expanded to increase database performance, reliability, quality management, and ease of use. Microsoft SQL Server 2010 database edition is a high-performance enterprise relational database management system with high reliability, ease of use, and other characteristics. SQL Server 2010 features more comprehensive specific features, as shown in Figure 3.

Therefore, this paper selected SQL Server 2010 for big data analysis; first of all, SQL Server 2010 version has been more mature; secondly, SQL Server is the management of large database use, that is, analysis of big data use; the use of the software is more appropriate; finally, SQL Server is more commonly used to analyze big data software.

#### 3.2.3. Oracle Big Data Analysis Research

**Figure 4:**
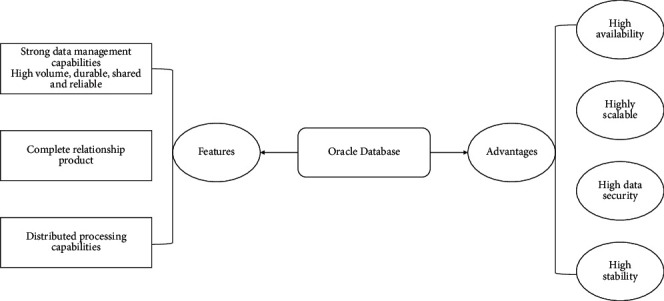
Features and advantages of Oracle database.

Oracle Database Management System is a relational database management system developed by the German company Oracle Software (Oracle in Chinese). It may also be another Microsoft database product that will be designed with distributed database design in mind as its most core feature. It will also be one of the most popular distributed C/S server architecture solutions or distributed B/S database architecture solutions used by Microsoft in the world. Compared with SQL Server database, the state of “doubt” is one of the most obvious and attractive performance advantages of the Oracle database parallel server model, which can achieve any one subquery decomposition into any multiple subqueries and then execute subroutines on two different server CPU processors, greatly improving the performance of multiprocessing systems, which should be a data trend with a great potential competitive advantage that is growing rapidly in the coming years. Oracle database also has many other significant advantages over: complete data storage management storage capacity; large data storage capacity, long persistence time, can be shared to ensure reliability. In addition, it has complete distributed management capability and is easy to operate. As shown in Figure 4.

## 4. Results and Discussion

### 4.1. Insurance Industry Development in the Era of Digital Economy

#### 4.1.1. The Growth Rate of Original Premium Income of Insurance Industry Returns to the Trend of Steady Growth

**Figure 5:**
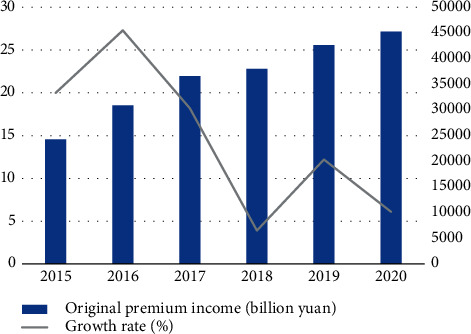
China's insurance original premium income and growth rate from 2015 to 2020.

According to the statistics of relevant departments, China's premiums show a growth trend, increasing from 2,428.3 billion yuan in 2015 to 4,525.7 billion yuan in 2020. However, the growth rate of original premium income experienced a brief decline from 18.2% to 3.9% from 2017 to 2018, due to the “1 + 4” series of documents issued by the former CIRC in 2017, which clearly defined the requirements of strengthening supervision, managing chaos, preventing risks, and serving the real economy in the insurance market, and various departments also launched relevant systems and measures, initiated special inspections, suspended investment-based risk business pilots, and heavily fined companies for violations. Since then, the business of insurance companies gradually matched the regulatory requirements, risks were released, and the insurance industry redeveloped, as shown in Figure 5.

#### 4.1.2. Policies Promote the Development of Insurance Digitalization

In recent years, China has been paying attention to the digital development of the insurance industry. In 2020, the CIRC pointed out that insurance companies should explore contactless underwriting and claims under the background of the epidemic and widely use digital technologies such as artificial intelligence and biotechnology to promote off-site investigation, and it is expected that, by 2022, the construction and governance of information security mechanisms and other aspects urge insurance companies to make rectification in data security and information docking; in the “2021 Digital Transformation Development Summit Forum” in April 2021, the CBIRC said it will further promote the digital transformation of insurance companies, clarify the risk bottom line of the transformation process of insurance companies, and formulate corresponding regulatory policies.

By combing through the national policy vein, it can be seen that the state advocates the insurance industry to use artificial intelligence, big data, biotechnology, and other technologies to empower insurance with technology, accelerate the process of digitalization and intelligence of insurance, promote the internal business effectiveness of the insurance industry, and reduce costs and increase efficiency. A major global insurance assessment company released an assessment report stating that the epidemic has accelerated the development of online insurance business. Insurance companies without the support of technology can hardly cope with future challenges. In this context, a series of policies and regulations promulgated by the State Council and CBIRC will provide a good opportunity for business transformation in the insurance industry.

#### 4.1.3. The New Crown Epidemic Catalyzes Insurers' Digital Upgrade

The new crown epidemic outbreak has affected the traditional offline agent model in the insurance industry, and according to McKinsey's “Life Insurance Marketing Transformation and Insurance Agent Workforce Empowerment 2020,” the first half of 2021, business growth in insurance types marketed through traditional offline channels, such as life insurance and auto insurance, has been hampered and slowed, while Internet platform insurance, such as health insurance, has continued its 2019 and 2020 growth momentum, climbing up.

**Figure 6:**
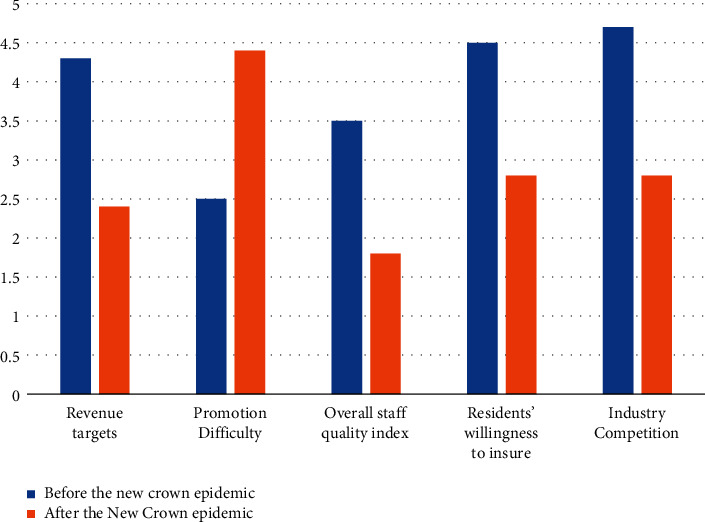
Comparison of various indicators of insurance companies before and after the epidemic.

All data show that the traditional insurance marketing method has reached a stage where it must be reformed. Influenced by factors such as the receding demographic dividend, the difficulty of offline promotion by insurance marketers leading to a sharp decline in revenue, and the gradual change in customer demand preferences for insurance products in the context of the epidemic, the sea of people tactic that has been going on for years in the insurance industry is destined to be unsustainable. The success of online transformation will be one of the key factors in the growth of premium income of each insurance type. If insurance companies do not carry out digital transformation in time and actively lay out new online tracks, it will have a great impact on insurance types that rely on traditional marketing models. The comparison of various indicators of insurance companies before and after the epidemic is shown in Figure 6.

#### 4.1.4. Capital Focuses on the Digitalization Process of Insurance

**Figure 7:**
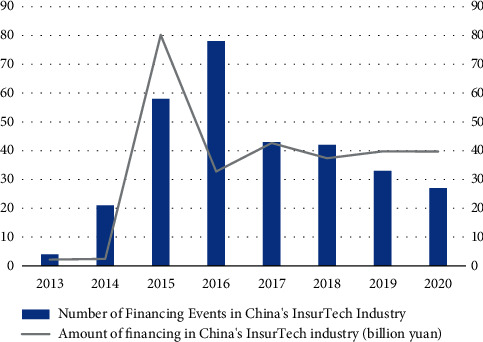
Statistics on the number of financing and amount of financing in China's InsurTech industry from 2013 to 2020.

The great prospect of InsurTech development in the digital economy era has attracted a large influx of venture capital. InsurTech ushered in the first wave of financing in 2015, with the number of financing pieces reaching 58 and the financing amount soaring to 8.017 billion yuan from 242 million yuan in 2014. The slowdown in the growth of the original premium income of China's insurance industry under the background of strong regulation in 2017 did not stop capital from enthusiastically chasing the InsurTech industry, and the InsurTech sector still absorbed a financing amount of 4.265 billion yuan in 2017. It is worth noting that although the InsurTech industry maintained a high capital fever from 2017 to 2020, with the financing amount floating above and below 4 billion yuan, the number of financing pieces started to decline from 2017 onwards instead and continued to go down in the following three years, proving that the proportion of small investments is decreasing, while the proportion of large investments is further increasing, and the industry is gradually maturing, as shown in Figure 7.

The use of insurance technology can eventually help the insurance industry to reduce operating costs, enrich product design, improve service experience, and cover a wider customer base with its business. In the context of the trade war between China and the US, the competition between China and the US will eventually turn into a battle of technology, and the only way to occupy a place in the global market is to use technology to promote the development of the industry. There are a lot of opportunities in this process, so the influx of capital is profitable, and it is foreseeable that the insurance technology industry will still be a hot target for capital in the future.

### 4.2. Challenges Facing the Insurance Industry in the Era of Big Data Economy

#### 4.2.1. Diversified Competition Pattern and Small- and Medium-Sized Insurance Companies Face Challenges

The competition pattern is evolving with the influx of various entities. According to the public data of the Insurance Industry Association, the competition among insurance companies in the same industry has been very intense, and there is also industry competition with banks, securities, and other financial institutions in the insurance industry. As the InsurTech industry continues to expand, more companies with financial strength and technical means will join the competition. Without the support of large companies, small- and medium-sized InsurTech companies will easily face the dilemma of lack of talent, technology, and capital, and even be unable to join the track. Large InsurTech companies actively develop new technologies, undertake industry data collection, and strengthen their own risk control capabilities and pricing levels, which will further aggravate adverse selection, resulting in a situation where the stronger the stronger and the weaker the weaker, which is like a disaster for the submature companies.

**Figure 8:**
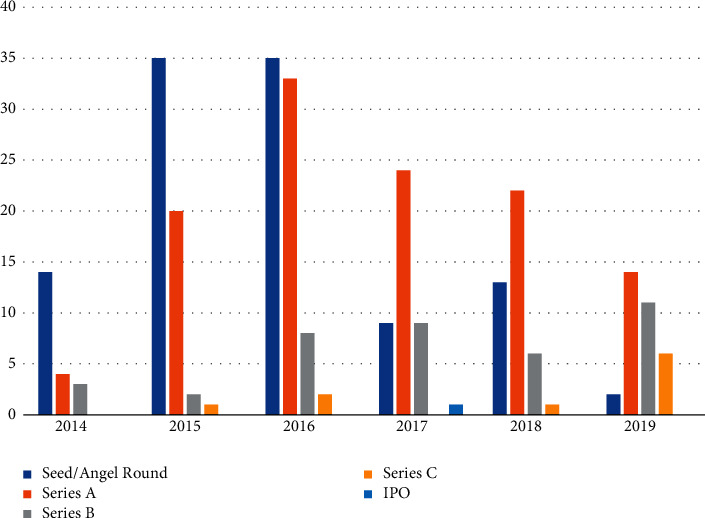
Distribution of InsurTech financing rounds in China, 2014–2019.

Financing winds have changed, and market competition has intensified. In 2015, the finance industry developed rapidly, and the number of transactions reached a new peak; however, the number of seed/angel round financing pieces began to show a downward trend in fluctuation and had dropped to 5% by 2019. The number of Series A rounds accounted for only 19% in 2014 and stabilized at around 40% from 2015 to 2019; the number of Series B rounds declined significantly compared to seed/angel and Series A rounds, accounting for only around 21% in 2017, the year with the largest share; Series C rounds varied widely from year to year, accounting for 18% in 2019 and 0% in 2014 and 2017. These investment evolutions have led to InsurTech companies being more difficult to obtain funding compared to before, with capital acting more cautiously and preferring to invest in projects in their infancy. But even if companies are able to secure Series A funding, moving from Series B to Series C or even Series D is still very challenging, judging by the relatively depleted Series B funding and the unstable Series C funding. Capital for the middle and late failure to achieve “independent blood” of the enterprise will choose to leave the field decisively. Digital technology is an important force in the development of the industry, but this force will intensify the elimination of the best and the worst among enterprises; some submature enterprises must race against time, or may not wait to show the technology in the industry will face a premature death, as shown in Figure 8.

#### 4.2.2. Insufficient Kinetic Energy for Industrial Transformation and Barriers to Technology Upgrade

There are barriers to data acquisition. The development of the insurance industry is to a certain extent constrained by big data. The digital era has accelerated the collection of information by insurers, and major insurers are also paying more and more attention to the collection of data. Large insurers such as Taiping and Taikang are gradually establishing their own independent health care organizations and developing artificial intelligence technologies to get closer to customers' lives and obtain richer data, while small- and medium-sized insurers are also taking this as a long-term development plan. However, at this stage, the collection and use of data by insurance companies is still in its infancy. The accumulation of data of traditional insurance companies mainly comes from the basic information of customers and claims data in their underwriting process, and a considerable part of the data has a large duplication, which can only be used as identity credentials and cannot be used as effective information to portray customer portraits, and cannot be used for in-depth risk mining or developing personalized needs. It is also difficult for traditional insurance companies to collect fragmented information and use it for modeling, which requires external support. Large companies that already have a large amount of user data, such as Meituan, Tencent, and Ali, prefer to improve their financial landscape by acquiring shares in insurance companies with relevant licenses, rather than serving the insurance industry. Insurance companies have very limited access to public data information from official channels, and data obtained from other nonprofessional third-party organizations may have inconsistent data standards. The existence of data barriers restricts the transformation and upgrading of insurance industry services and hinders the deep integration of insurance and modern information technology:

 
*Lack of Composite Talents*. Although, in China, the supply of talent in the financial industry is much greater than the demand for talent, but from the perspective of China's entrepreneurial innovation in the financial sector, most of the innovation and entrepreneurship in the financial sector is still biased towards the sales industry, and there is still relatively little research on specific research and development and computing algorithms. Therefore, the platform of sales, which can better solve the problem of sales, can solve the problem of insurance sales in China and solve the problem of the status quo of online sales, which can make it more convenient for consumers to choose the insurance products they need. According to the data published by the CBIRC, Internet premium income reached 269.63 billion yuan in 2019, up 42.8% year-on-year. The change in the marketing model also means a shift in the demand for talent in the insurance industry. The digital era has made insurance companies more hungry for “insurance + technology” composite talents. In 2019, PwC pointed out in its report that there are a large shortage of financial engineering technical posts and a shortage of more than one million financial technology positions, and talents who can combine technology and finance are the objects of competition for major companies. From the recruitment status of major insurance companies, each insurance company attaches great importance to the development of the field of insurance technology. The chief human resources executive of Ping An Group said in an interview that the technology business segment of Ping An Group has onboarded 6,000 people and will further expand the introduction of talents in the future. From a macro point of view, whether the insurance industry can play the advantages brought by insurance technology in the future depends on the application of talents to new technologies and their adaptation to emerging technologies, while there is still a relative lack of insurance technology talents in China.
 
*Technology Penetration Barrier*. The application of digital technology will undoubtedly greatly improve the efficiency of the insurance industry, reduce operating costs, and inject new blood into the development of the insurance industry, but for most insurance companies, the application of digital technology is still in its infancy, and there are certain loopholes in both the technology itself and the technology application environment. As of now, the application of big data technology is the highest in each exhibition environment, followed by artificial intelligence, while blockchain technology, which has the most potential value for application, is still in the stage of pending development. 5G technology, as an auxiliary technology for the above-mentioned technologies, has not yet achieved the full coverage of its base station construction, and the main coverage users are still large city groups. The ultimate goal of digital technology application in the insurance industry is to create an integrated and convergent business development model with omnichannel and omniplatform, which requires joint research and development, investment, and cooperation among major insurance companies, and only a few insurance companies applying related technologies cannot achieve the goal of integrated business development. However, for traditional insurance companies that have already acquired a certain user base and have a model business process, it is still doubtful how much money they are willing to invest in RD and infrastructure construction of new technologies and whether they are willing to share technologies after considering the cost-to-benefit ratio. At present, it is obvious that the technology application status of major insurance companies still cannot meet the requirements of digital technology and insurance industry integration, and there are still some obstacles for digital technology to penetrate the insurance industry.

#### 4.2.3. Changes in the Macro Environment and Increased Business Risks

The regulatory environment is tightening, and policies are gradually being established. In June 2020, China's central bank as well as the CBIRC and other departments jointly issued the latest notice on regulating the insurance industry, the financial industry. It requires that the maintenance of sales records and the regulation of the sales process must be completed by October 2020, further strengthening the regulation of the sales process for back checking when necessary, and specific sales scenarios require audio and video recording, further clarifying the requirements for the sale of Internet products. For some Internet insurance companies or large insurance companies, the capital and RD capabilities enable them to complete the retrospective work rectification earlier, but for other more insurance companies, it is still not a small amount of work. In January 2021, the CBIRC, together with the Central Bank, the Ministry of Finance, and other departments, further improved and issued a further standardized approach for insurance and other intermediaries. This approach requires insurance and other financial institutions to be completed within a year, to be able to complete the relevant records in a timely manner, the relevant sales aspects of the process of tracing, and complete records of the financial business personnel situation of the information technology construction, and to achieve business docking with the insurance company to generate data conditions that meet the regulatory conditions. However, the current situation, in general, most of the companies still take the offline docking approach, and did not really take the Internet online sales approach to take online docking. From the above-mentioned series of regulatory measures, it can be seen that the insurance regulators are gradually implementing regulatory measures to regulate the process of insurance digitization, maintain market order, and gradually eliminate some “small and messy” business entities.

## 5. Conclusion

According to the definition of big data technology issued by relevant Chinese research institutes, big data technology is defined as a technology that can be used for digitalization. Big data technology is defined as follows: big data technology is an important productivity factor of digitalization in the network era; big data technology is based on network technology, statistical technology, and mathematical technology; big data technology, as a carrier of network technology, should be integrated with various industries and technologies other than contemporary ones in order to achieve better informatization and industrialization. . In the context of the new crown epidemic in 2020, China's big data technology continues to grow steadily, with a scale of 39.2 trillion yuan, accounting for 38.6% of GDP. At the same time, China's big data technology was included in the development of China's plan to promote the comprehensive development of big data, the development of big data into all walks of life, the process of China's digitalization, and the building of digital power. This paper investigates the risk constraint in the context of big data in the context of rapid information technology development and optimizes the benign interaction mechanism of insurance management based on distorted risk metrics. The current state of insurance risk is investigated through a thorough discussion of theories in this paper and research methods such as distorted risk metrics and SQL Server database management. It has been discovered that the overall situation of the insurance industry's development in the digital economy is not optimistic and so on. The following suggestions are made.

At the level of traditional insurance companies, the first is to accelerate digital transformation and strengthen digital construction. The second is to improve the talent cultivation mechanism and pay attention to the cultivation of composite talents. At the level of new insurance enterprises, the first thing is to maintain the advantages and actively innovate. The second is to be open and inclusive, and mutually beneficial. At the government level, the first is to establish a multilevel subsidy system to support the growth of insurance science and technology companies, and the second is to improve the incentive system to encourage the digital transformation of traditional insurance enterprises.

## Data Availability

The dataset is available upon request.
